# Sleep and circadian hygiene practices association with sleep quality among Brazilian adults

**DOI:** 10.1016/j.sleepx.2023.100088

**Published:** 2023-10-07

**Authors:** Laura Castro-Santos, Márcia de Oliveira Lima, Anny Kariny Pereira Pedrosa, Renan Serenini, Risia Cristina Egito de Menezes, Giovana Longo-Silva

**Affiliations:** a Research Group ‘Chronobiology, Nutrition, and Health', Faculty of Nutrition, Federal University of Alagoas, Brazil; b Faculty of Economics, Sapienza University of Rome, Via del Castro Laurenziano, Rome, Italy

**Keywords:** Sleep, Sleep quality, Chronobiology, Sleep hygiene

## Abstract

**Objective:**

To investigate the association of sleep and circadian hygiene practices (sleep-promoting and sleep-disturbing behaviors) with sleep quality indicators.

**Methods:**

Participants (n = 2050; 18–65 y) were part of virtual population-based research. Logistic regression models were fitted to assess differences in the OR (95% CI) of poor quality with sleep-promoting/disturbing practices (time-of-day of exercise, pre-bedtime routine, naps, electronic devices with illuminated screens, caffeine and alcohol consumption, and smoking). Linear regression analyses evaluated differences in sleep duration, latency, and awakenings associated with the same variables. Restricted cubic splines were used to study the shape of the association of screen time before bed with sleep duration, latency, and awakenings. Analyses were adjusted for age, sex, region, marital status, educational level, evening diet quality, and BMI.

**Results:**

Evening use of electronic devices with illuminated screens showed a negative effect on all sleep parameters. Reporting dinner as the largest meal and evening caffeine consumption was associated with shorter sleep duration and longer sleep latency. Smokers had higher odds of longer latency. A protective effect of morning exercises was demonstrated on sleep quality, latency, and awakenings. Alcohol consumers presented lower odds of poor quality and lower frequency of awakenings. Pre-bedtime practices showed no or negative effect on sleep outcomes.

**Conclusions:**

Recommendations to promote sleep quality and prevent sleep-related problems, with corresponding circadian health benefits, should include engaging in regular exercise, preferably in the morning, and avoiding naps, heavy meals close to bedtime, caffeine, smoking, and evening screen exposure.

## Highlights

• This study was carried out using data from the first Brazilian survey on chronobiological aspects related to sleep, nutrition, and health.
• A protective effect of morning exercises was demonstrated on sleep quality and sleep latency.
• Screen use before bed was associated with sleep duration, sleep latency and nocturnal awakenings.
• Nappers presented decreased nocturnal sleep duration and increased awakenings.
• Dinner as the largest meal and caffeine consumption associated with sleep duration, latency and quality.

## 1 Introduction

In modern society, sleep-related problems have become an important public health problem and frequent complaints in clinical practice. It is estimated that about 1/3 of the world's population has insufficient sleep [1], with chronic insomnia being the most common sleep disorder, with a worldwide prevalence of around 10% [2].

Sleep disorders or disrupted sleep from lifestyle choices, environmental conditions, or other medical issues can lead to significant morbidity and can contribute to or exacerbate medical conditions [[3], [4], [5]]. The consequences of sleep conditions take a toll on nearly every key indicator of public health: mortality, morbidity, performance, accidents, and injuries, functioning and quality of life, family well-being, and healthcare utilization [6].

Global public health concern over sleep has increased demand for sleep promotion strategies, and, in addition to the recognized sleep hygiene, which integrates all guidelines and strategies for prevention and/or aid in the adjuvant treatment of sleep disorders [7], a new concept was recently proposed by chronobiologists: circadian hygiene [8].

Sleep hygiene education is intended to provide information about lifestyle and environmental factors that may either interfere with or promote better sleep. Regular exercise (but not too close to bedtime), regular bed and/or wake times, having a prebedtime ‘wind-down’ routine, avoiding/limiting stimulants (e.g., caffeine) for several hours before bedtime, avoiding naps, and limiting alcohol intake are examples of behaviors included on this customized list to enhance sleep quality [4,9,10].

Besides these recommendations, circadian hygiene focuses on the synchronization of the circadian biological rhythms with the 24-h cycle, to prevent the misalignment between the circadian pacemaker and behavioral/environmental cycles [8]. Insufficient sleep and circadian misalignment, which have received increased research attention in the past 10 years, lead to changes in energy-regulating hormones, impair cognitive performance, and predispose individuals to poor metabolic health [[11], [12], [13]].

Because the circadian system of both nocturnal and diurnal organisms is most sensitive to light during the biological night, avoiding evening use of electronic devices with illuminated screens close to usual bedtime, through the night, and about 1 h after the usual wake-up time has been highlighted to improve sleep quality [6,14,15].

However, although epidemiologic and experimental research generally supported an association between sleep hygiene and sleep, the direct effects of individual recommendations on sleep remain largely untested in the general population [9,16,17] Thus, the effectiveness of sleep hygiene, especially in nonclinical populations is discussed [4] and further knowledge must be acquired to improve education not only for individuals but also to embrace reflections and changes in policies promoting healthy lifestyle habits in a technological society with work and cultural shifts that are often inconsistent with recommendations for healthy sleep [8].

Therefore, to assist in filling this gap, and contribute to debates and reflections on the applicability of sleep hygiene in the general population, using data from the first Brazilian survey with a chronobiological focus on sleep, nutrition, and health, our study aimed to investigate the association of sleep-promoting and sleep-disturbing behaviors (time-of-day of exercise, pre-bedtime practices, naps, evening use of electronic devices with illuminated screens, evening caffeine and alcohol consumption, and smoking) with sleep quality indicators (duration, self-perception of sleep quality, latency, and awakenings).

## 2 Material and methods

### 2.1 Study design and population

This study was carried out with data from the first and second stages of the SONAR-Brazil Survey, which aims to investigate chronobiological aspects related to sleep, food, and nutrition in Brazilian adults. This is exploratory, population-based research, with data collection exclusively in a virtual environment. Participants were adults, non-pregnant, aged between 18 and 65 years, born and residing in all regions of Brazil (n = 2140). After excluding participants who declared being shift workers (n = 90) the final sample totalized 2050 non-pregnant Brazilian adults.

Considering a large population, to estimate population proportions with a confidence level of 95% and a margin error of 5% we defined, a priori, a minimum sample size of 385 valid questionnaires. However, the sample size remained open, and the efforts were directed to increase as maximum as possible to minimize the error margin. The final sample of 2050 guarantees proportion estimates with a 95% confidence level and a margin of error lower than 4%. All data collection procedures have been conducted according to the Declaration of Helsinki and approved by the Committee of Research Ethics.

Recruitment took place between August 2021 and September 2022 and data were collected using a Google Form. By clicking on the research link, the volunteer respondents were directed to informed consent and, only after indicating their consent to participate in the study, they were directed to the questionnaire, made up of four blocks: characterization, health, and lifestyle, sleep characteristics, eating and sleeping schedules. The generated responses were automatically stored in spreadsheets compatible with Microsoft Office Excel and later exported to the statistical software STATA 13 statistical software [Stata Corporation] for statistical analyses. The link to the online questionnaire was disseminated in several ways: referral of health professionals' reports in newspapers/magazines, advertisements on social media platforms, research institutes, health fairs, events scientific journals, and electronic pages addressing the research participants, to increase research visibility and, consequently, data collection.

### 2.2 Sleep traits

In the questionnaire block about ‘eating and sleeping schedules’, the participants were informed: ‘In this section, we want to know your routine on weekdays/workdays and weekends/free days’. The following questions were used to measure usual sleep and wake times: ‘Considering your habits during the last month, on a typical weekday (or weekend)’ 1. What time do you wake up? 2. What time do you sleep? Responses were in 30-min increments.

Sleep duration (in hours) was calculated as the difference between bedtime and wake-up timing [18,19]. We also calculated the midpoint of sleep on weekdays and weekends, defined as the middle time point between bedtime and wake-up timing [18,20].

The average weekly sleep duration, wake-up time, bedtime, and the midpoint of sleep were calculated as follows: [(5 × sleep duration/wake-up time/bedtime/midpoint of sleep on weekdays) + (2 × sleep duration/wake-up time/bedtime/midpoint of sleep on weekends)]/7 [18,19].

Social Jetlag (SJL) was calculated by the absolute difference between the mid-sleep time on weekends/free days and the mid-sleep time on weekdays/workdays [20,21].

We adopted the “midpoint of sleep on free days corrected for sleep extension on free days (MSFsc)” as an indicator of chronotype, which is proposed to clean the chronotype of the confounder sleep debt [21]. For participants whose sleep duration on free days was longer than work days, the midpoint was calculated as follows: [bedtime on free days + (sleep duration on free days/2)]. For participants whose sleep duration on free days was shorter than work days, due to the sleep debt accumulated over the workweek, the corrected midpoint of sleep was applied, and calculated as follows: [bedtime on free days + (weekly average sleep duration/2)]. For more details on the methodology see Refs. [20,21].

Sleep latency was investigated by asking: ‘During the past month, how long (in minutes) has it usually taken you to fall asleep each night?’ and nocturnal awakenings by: “How many times do you wake up during the night, after sleep onset?”.

Self-perception of sleep quality was investigated based on the question: “How do you rate the quality of your sleep?”, with the possible answers: very good, good, poor, very poor. We considered poor sleep quality for those who answered poor or very poor.

### 2.3 Sleep and circadian hygiene practices

#### 2.3.1 Time-of-day of exercise

The physical exercise practice and time-of-day were investigated based on the following questions: 1. “Do you practice physical exercise (of moderate or vigorous intensity)?” (e.g.: walking, treadmill walking, bodybuilding, hydro gymnastics, gymnastics in general, swimming, martial arts and fighting, cycling, volleyball/football, dance, running, treadmill running, aerobics, soccer/futsal, basketball, and/or tennis); 2. “Which time-of-day do you usually exercise?”, and possible answers were: morning (5:00 to 12:00), afternoon (12:00 to 18:00), and/or evening (after 18:00).

#### 2.3.2 Pre-bedtime routine

Participants were asked about a pre-bedtime routine aimed to promote sleep, including: (i) integrative therapies (e.g., meditation, aromatherapy, color therapy, homeopathy, music therapy, flower remedies, yoga), (ii) drinking soothing teas, (iii) reading books (iv) dimming environment lights in the evening.

#### 2.3.3 Naps

Participants were asked about taking naps during the day on weekdays (or weekends) and, if so, about its usual timing (hh:mm) and duration (min). We considered nappers those who reported napping on weekdays and/or weekends. The average weekly nap duration and timing were calculated as follows: [(5 × nap duration/nap timing on weekdays) + (2 × nap duration/nap timing on weekends)]/7.

#### 2.3.4 Evening use of electronic devices with illuminated screens

Evening use of electronic devices with illuminated screens was measured by the questions: 1. “Right before bedtime, how long do you spend using electronic devices (e.g., TV, computer, tablet, cell phone)?” (min). 2. “Do you use electronic devices as a strategy to help you fall asleep?” (yes or no); and 3. “Do you sleep with any screen turned on in the bedroom/sleeping environment? (e.g., TV, computer, tablet, cell phone)?” (yes or no).

#### 2.3.5 Evening diet, caffeine and alcohol consumption, and smoking

Food consumption was investigated using a food frequency questionnaire comprising 19 food categories, for which participants selected the frequency of weekly consumption: ‘never’, ‘sometimes (1–3 days/week)’, ‘almost always (4–6 days/week)’ or ‘always (6–7 days/week)’. Sequentially, participants reported the daily frequency and timing of consumption, with responses in the intervals between 6:01–9:00, 9:01–12:00, 12:01–15:00, 15:01–18:00, 18:01–21:00, 21:01–00:00, after 00:00.

We evaluated the evening diet quality, considering the frequency of food consumption after 18:00, based on the Food Guide for the Brazilian Population, as proposed in previous studies [18,19].

Food markers for healthy eating (fresh fruits; vegetables and/or legumes; beans, chickpeas, lentils and/or peas; milk and/or dairy products; eggs; meats; fish) received increasing scores (never = 0; sometimes = 1; almost always = 2; always = 3), while unhealthy eating markers (snacks; chocolate; fried snacks; instant noodles, packaged snacks or crackers; fast food; hamburger and/or sausages; coffee; soda cola-based; sweetened beverages; mate or black tea; guarana powder; alcoholic beverages), decreasing (never = 3; sometimes = 2; almost always = 1; always = 0). From the sum of the scores of each food category, the total score of the Diet Quality Index (DQI) was obtained, which could range from 0 to 57 points, with a higher score suggestive of a higher frequency of consumption of healthier foods and lower frequency of consumption of unhealthy foods. Sequentially, from the available scores, it was created tertiles for diet quality classification: 1st tertile – low quality (21–34 points); 2nd tertile – intermediate quality (35–38 points), and 3rd tertile – good quality (39–47 points).

Furthermore, we analyzed evening consumption (after 18:00) of alcohol and caffeine foods/beverages, which included coffee, soda cola-based, mate or black tea, and/or guarana powder.

Participants were also asked: “What is your largest meal of the day?”, which refers to the meal in which participants consume the greatest amount of calories, and possible responses were: Breakfast, Lunch, Dinner, or None [22,23].

Some other details of the survey questionnaire concerning other covariates including sex, age, region, education, marital status, and smoking were included in our study.

### 2.4 Statistical analyses

To assess differences between sleep duration (<7 or ≥7 h) night [24] self-perception of sleep quality (good or poor), in their characteristics, and sleep hygiene behaviors (sleep-promoting and sleep-disturbing practices), the student's *t* test (for continuous variables) and the chi-square test (for categorical variables) were performed.

Logistic regression models were fitted to assess differences in the ORs and 95% CIs of poor sleep quality indicators (outcomes) with the continuous and categorical sleep-promoting and sleep-disturbing practices.

Linear regression analyses evaluated differences in sleep duration, sleep latency, and nocturnal awakenings (outcomes) associated with the same sleep-promoting and sleep-disturbing practices. Regression coefficient (β) and 95% CIs were calculated for the unadjusted and adjusted models.

Moreover, restricted cubic splines were also used to study the shape of the association of screen time before bed with sleep duration, latency, and nocturnal awakenings.

All the multiple analyses were adjusted for potentially confounding variables: age, sex, region, marital status, educational level, evening diet quality, and BMI. A P-value of ≤0.05 was considered statistically significant.

## 3 Results

A total of 2050 adults [age: 34 y (range 18, 65); 73% women, 39% with stable union, 70% college graduated or above] were enrolled (Table 1). Among all participants, the averages of sleep duration, latency, and awakenings were, respectively 7.8 h, 34min and 1/night and 21% were short sleepers (<7 h), and 30% reported poor sleep quality (Table 1). Among nappers (51% of the total), more than half slept for more than 30 min, and 15% after 15:00.

**Table 1:** Characteristics and sleep hygiene practices of participants by sleep duration and self-perception of sleep quality (n = 2050).

Variables	Total	Sleep Duration			Sleep Quality		
		<7 h	≥7 h	P value*	Poor	Good	P value*
**Total,%**	2.050 (100.00)	445 (21.71)	1605 (78.29)		613 (29.9)	1437 (70.1)	
**Sex,% female**	1.498 (73.07)	285 (64.04)	1213 (75.58)	**<0.001**	448 (73.08)	1050 (73.07)	0.99
**Age, y**	34.32 ± 11.59	34.61 ± 11.29)	34.24 ± 11.67)	0.55	35.29 ± 11.55	33.91 ± 11.58	**0.01**
**Brazil macro-region**							
*North****, %***	134 (6.54)	35 (7.87)	99 (6.17)	**<0.001**	44 (7.18)	90 (6.26)	**0.01**
*North East****, %***	720 (35.12)	191 (42.92)	529 (32.96)		238 (38.83)	482 (33.54)	
*Southeast,****%***	810 (39.51)	159 (35.73)	651 (40.56)		230 (37.52)	580 (40.36)	
*South****, %***	224 (10.93)	29 (6.52)	195 (12.15)		48 (7.83)	176 (12.25)	
*Midwest****, %***	162 (7.90)	31 (6.97)	131 (8.16)		53 (8.65)	109 (7.59)	
**Marital Status**							
Married/Living with Partner**, %**	793 (38.68)	160 (35.96)	633 (39.44)	0.18	240 (39.15)	553 (38.48)	0.77
**Education level**							
*Less than High School, %*	34 (1.66)	13 (2.92)	21 (1.31)	**0.02**	14 (2.28)	20 (1.39)	0.26
*High school, %*	572 (27.90)	133 (29.89)	439 (27.35)		163 (26.59)	409 (28.46)	
*College graduate or above, %*	1444 (70.44)	299 (67.19)	1145 (71.34)		436 (71.13)	1008 (70.15)	
**BMI, kg/m**^**2**^	25.14 ± 5.03)	25.82 ± 5.25	24.95 ± 4.95	**0.001**	25.82 ± 5.48	24.85 ± 4.80	**0.0001**
***Sleep Traits***							
***Sleep Duration, h***	7.79 ± 1.17	6.20 ± 0.69	8.23 ± 0.85	**<0.001**	7.51 ± 1.36	7.91 ± 1.05	**<0.001**
***Latency, min***	33.47 ± 36.69	39.81 ± 44.65	31.71 ± 33.97	**<0.001**	55.89 ± 48.00	23.91 ± 25.16	**<0.001**
***Awakenings, number/night***	1.3 ± 1.23	1.40 ± 1.34	1.27 ± 1.20	0.04	2.00 ± 1.39	0.99 ± 1.02	**<0.001**
***Social jet lag, h***	0.35 ± 0.73	0.50 ± 0.87	0.31 ± 0.68	**<0.001**	0.41 ± 0.79	0.33 ± 0.70	**0.01**
***Sleep-promoting practices***							
**Morning Physical Exercise, %**	675 (32.93)	140 (31.46)	535 (33.33)	0.45	155 (25.29)	520 (36.19)	**<0.001**
**Dim environment lights in the evening, %yes**	836 (40.78)	171 (38.43)	665 (41.43)	0.25	278 (45.75)	558 (38.83)	**0.006**
**Read books before bed, %yes**	326 (15.90)	75 (16.85)	251 (15.64)	0.53	118 (19.25)	208 (14.47)	**0.007**
**Drink Soothing tea before bed, %yes**	443 (21.61)	96 (21.57)	347 (21.62)	0.98	178 (29.04)	265 (18.44)	**<0.001**
**Integrative therapies before bed, %yes**	346 (16.88)	69 (15.51)	277 (17.26)	0.38	126 (20.55)	220 (15.31)	**0.004**
***Sleep-disturbing practices***							
**Evening Physical Exercise, %**	573 (27.95)	123 (27.64)	450 (28.04)	0.86	159 (25.94)	414 (28.81)	0.18
**Nap**							
*Yes*	1045 (50.98)	242 (54.38)	803 (50.03)	0.10	315 (51.39)	730 (50.80)	0.80
*Duration (min)*	21.58 ± 34.47	26.34 ± 46.48	20.25 ± 30.16	**0.001**	24.33 ± 42.24	20.42 ± 30.54	**0.02**
*Duration >*30min*, %*	556 (55.38)	140 (59.32)	416 (54.17)	0.16	188 (62.88)	368 (52.20)	**0.002**
*Timing, hh:mm*	13.81 ± 1.80	13.55 ± 1.71	13.89 ± 1.82	0.07	13.78 ± 2.28	13.82 ± 1.53	0.80
*After 15:00, %*	72 (15.62)	16 (13.91)	56 (16.18)	0.56	27 (18.75)	45 (14.20)	0.21
**Electronic devices with illuminated screens**							
*Time before bed (min)*	61.70 ± 71.45	72.66 ± 90.77	58.66 ± 64.79	**<0.001**	74.33 ± 75.19	56.31 ± 69.12	**<0.001**
*Time before bed ≥2 h, %*	430 (20.98)	112 (25.17)	318 (19.81)	**0.01**	171 (27.90)	259 (18.02)	**<0.001**
*Use of screens to fall asleep, %yes*	665 (32.44)	145 (32.58)	520 (32.40)	0.94	239 (38.99)	426 (29.65)	**<0.001**
*Sleep with screens turned on, %yes*	806 (39.32)	192 (43.15)	614 (38.26)	0.06	290 (47.31)	516 (35.91)	**<0.001**
**Evening Eating**							
*Dinner is the largest meal, %yes*	230 (11.22)	68 (15.28)	162 (10.09)	**0.002**	91 (14.85)	139 (9.67)	**0.001**
*Caffeine consumption, %*	1183 (57.71)	264 (59.33)	919 (57.26)	0.43	387 (63.13)	796 (55.39)	**0.001**
*Alcohol consumption, %*	996 (48.59)	201 (45.17)	795 (49.53)	0.10	275 (44.86)	721 (50.17)	**0.02**
**Smoking***, %yes*	124 (6.05)	37 (8.31)	87 (5.42)	**0.02**	39 (6.36)	85 (5.92)	0.69

Abbreviations: BMI, body mass index.

Values are shown as means ± SDs or numbers (percentages). P values are derived from the student's t-test (for continuous variables) and from the chi-square test (for categorical variables).

Significant P-values ≤0.05 are shown in bold.

Table 1 also presents the characteristics of the studied participants, depending on sleep duration (<7 or ≥7 h) and sleep quality (good or poor).

Participants with short sleep duration and poor sleep quality reported longer naps (26 min versus 20 min and 24 min versus 20 min, respectively) when compared to the groups with non-short sleep duration and good sleep quality, respectively (All P < 0.05) (Table 1).

Incorporating pre-bedtime sleep-promoting practices (integrative therapies, dimming environment lights in the evening, reading books, drinking soothing tea) was more frequent among those with poor sleep quality, in comparison to participants with good sleep quality (p < 0.01). Furthermore, evening alcohol consumption was reported by 49% of the participants, and this percentage was significantly higher among those with good sleep quality perception, compared to the group with poor perception of sleep quality (50% versus 45%, p = 0.02) (Table 1).

Regarding evening use of electronic devices with illuminated screens, its use was longer among participants with short sleep duration (difference of 14 min) and poor sleep quality (difference of 18 min), compared to non-short and good sleepers’ groups, respectively. Also, using electronic devices as a strategy to fall asleep, and sleeping with screens turned on were more prevalent among those with poor sleep quality (p < 0.001) (Table 1).

Participants with short sleep duration and poor sleep quality more frequently reported dinner as the largest meal and the differences between the groups were significant. Also, compared to the group with good sleep quality, caffeine consumption after 18:00 was more frequent among participants with poor sleep quality (63% versus 55%) (P < 0.01) (Table 1).

Few participants were smokers (6%) and in group comparisons, the frequency was higher among those with shorter sleep duration (Table 1).

**Fig. 1:**
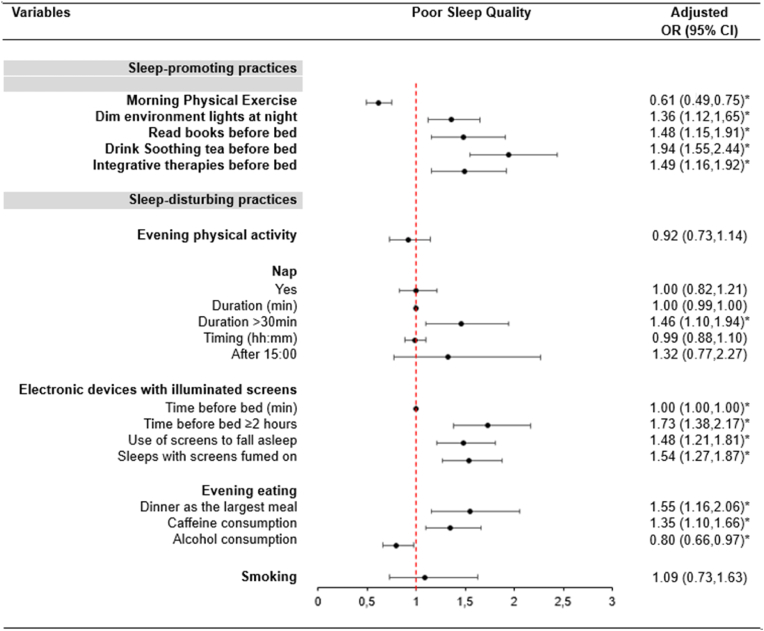
Multiple logistic models examining sleep-promoting and sleep-disturbing practices associated with self-perception of sleep quality. Data are presented as OR (95% CI) and the models are adjusted for age, sex, region, marital status, educational level, evening diet quality, and BMI. Abbreviations: OR, Odd Ratio; CI, Confidence Interval. * P-values derived from multiple logistic models are significant (≤0.02).

The results of multiple logistic analyses adjusted for age, sex, region, marital status, educational level, evening diet quality, and BMI are shown in Fig. 1.

Among sleep-promoting practices, morning physical exercise decreased the odds of self-perceived poor sleep quality (OR = 0.61; 95% CI = 0.49, 0.75; p < 0.001), while the other behaviors demonstrated negative effects on outcomes. Participants who dimmed the environment lights in the evening, read books, drank soothing tea, and/or incorporated integrative therapies before bed, presented higher odds of poor sleep quality (Fig. 1).

Regarding sleep-disturbing practices, napping for more than 30 min was associated with 46% higher odds (95% CI = 1.10, 1.94; p = 0.009) of self-perceived poor sleep quality (Fig. 1). Using electronic devices with illuminated screens for ≥2 h increased by 73% the odds (95% CI = 1.38, 2.17; p < 0.001) of poor sleep quality. Also, participants who reported using electronic devices as a strategy to fall asleep had higher odds of poor sleep quality (OR = 1.48; 95% CI = 1.21, 1.81; p < 0.001). Finally, sleeping with screens turned on in the bedroom increased by 54% the odds (95% CI = 1.27, 1.87; p < 0.001) of reporting poor sleep quality (Fig. 1).

Those who reported dinner as the largest meal (OR = 1.55; 95% CI = 1.16, 2.06; p = 0.003) and consumed caffeine after 18:00 had 35% higher odds (95% CI = 1.10, 1.66; p = 0.003) presented higher odds of poor sleep quality (Fig. 1).

Evening alcohol consumers presented lower odds of poor sleep quality (OR = 0.80; 95% CI = 0.66,0.97; p = 0.02) (Fig. 1).

Table 2 shows the multiple linear regression models. After adjusting for age, sex, region, marital status, educational level, evening diet quality, and BMI, there was a reduction in sleep latency (β = −7.41; 95% CI = −10.82, −4.00; p < 0.001) and in number of awakenings (β = −0.19; 95% CI = −0.31, −0.08; p = 0.001) among participants who exercised in the morning. The pre-bedtime behaviors were associated with longer sleep latency [dimming environment lights in the evening (β = 6.61; 95% CI = 3.38, 9.84; p < 0.001), reading books (β = 5.80; 95% CI = 1.43, 10.16; p = 0.009), drinking soothing tea (β = 11.08; 95% CI = 7.19, 14.96; p < 0.001) and incorporating integrative therapies (β = 5.65; 95% CI = 1.37, 9.93; p = 0.01)]. Among those who drank soothing tea, awakenings were also higher (β = 0.15; 95% CI = 0.02, 0.27; p = 0.02) (Table 2).

**Table 2:** Multiple linear regression models examining sleep-promoting and sleep-disturbing practices associations with sleep duration, latency, and nocturnal awakenings. Data are presented as β coefficient (95% CI) and the models are adjusted for age, sex, region, marital status, educational level, evening diet quality, and BMI.

	Sleep Duration (hour)		Sleep Latency (min)		Nocturnal Awakenings (n/night)	
Sleep Hygiene	Adjusted β (95% CI)	P value*	Adjusted β (95% CI)	P value*	Adjusted β (95% CI)	P value*
**Sleep-promoting practices**						
**Morning Physical Exercise**	−0.04 (−0.15,0.06)	0.43	−7.41 (−10.82,-4.00)	**<0.001**	−0.19 (−0.31,-0.08)	**0.001**
**Dim environment lights in the evening**	0.04 (−0.05,0.14)	0.37	6.61 (3.38,9.84)	**<0.001**	0.05 (−0.05,0.16)	0.30
**Read books before bed**	−0.14 (−0.27,-0.00)	**0.04**	5.80 (1.43,10.16)	**0.009**	0.00 (−0.13,0.14)	0.94
**Drink Soothing tea before bed**	−0.07 (−0.20,0.04)	0.22	11.08 (7.19,14.96)	**<0.001**	0.15 (0.02,0.27)	**0.02**
**Integrative therapies before bed**	0.00 (−0.13,0.13)	0.98	5.65 (1.37,9.93)	**0.01**	0.09 (−0.04,0.24)	0.17
**Sleep-disturbing practices**						
**Evening Physical Exercise**	0.02 (−0.08,0.13)	0.67	0.82 (−2.75,4.40)	0.65	−0.02 (−0.14,0.89)	0.63
**Nap**						
Yes	−0.10 (−0.21,-0.00)	**0.03**	−2.00 (−5.18,1.17)	0.21	0.14 (0.04,0.25)	**0.005**
Duration (min)	−0.00 (−0.00,0.00)	0.08	0.01 (−0.02,0.06)	0.46	0.00 (−0.00,0.00)	0.10
Duration >30min	0.00 (−0.14,0.14)	0.97	7.28 (2.99,11.57)	**0.001**	0.04 (−0.11,0.19)	0.60
Timing (hh:mm)	0.05 (−0.00,0.12)	0.07	−0.70 (−2.71,1.29)	0.48	0.04 (−0.01,0.10)	0.17
After 15:00	−0.02 (−0.34,0.30)	0.90	3.27 (−6.71,13.26)	0.52	0.13 (−0.18,0.44)	0.41
**Electronic devices with illuminated screens**						
Time before bed (min)	−0.00 (−0.00, −0.00)	**0.01**	0.10 (0.08,0.12)	**<0.001**	0.00 (0.00,0.00)	**0.01**
Time before bed ≥2 h	−0.12 (−0.25,-0.00)	**0.04**	16.98 (13.14,20.82)	**<0.001**	0.06 (−0.05,0.19)	0.29
Use of screens to fall asleep	−0.04 (−0.15,0.06)	0.40	14.42 (11.07,17.77)	**<0.001**	0.11 (0.00,0.22)	**0.04**
Sleep with screens turned on	−0.08 (−0.18,0.02)	0.12	9.08 (5.84,12.32)	**<0.001**	0.20 (0.09,0.30)	**<0.001**
**Evening Eating**						
Dinner is the largest meal	−0.20 (−0.36,-0.04)	**0.01**	9.39 (4.35,14.43)	**<0.001**	0.06 (−0.10,0.23)	0.45
Caffeine consumption	−0.16 (−0.26,-0.05)	**0.003**	3.66 (0.31,7.00)	**0.03**	0.08 (−0.02,0.19)	0.11
Alcohol consumption	0.06 (−0.04,0.16)	0.24	−0.37 (−3.60,2.85)	0.82	−0.15 (−0.26,-0.05)	**0.003**
**Smoking**	−0.09 (−0.30,0.11)	0.38	12.38 (5.66,19.10)	**<0.001**	0.11 (−0.10,0.33)	0.31

Abbreviation: CI, Confidence Interval.

β values reflect the difference in sleep duration (hours), latency (min), and nocturnal awakenings (n/night) by each practice.

Significant P-values ≤0.05 are shown in bold.

Regarding sleep-disturbing practices, napping (β = −0.10; 95% CI = −0.21, −0.00; p = 0.03), using screens ≥2 h before bed (β = −0.12; 95% CI = −0.25, −0.00; p = 0.04), reporting dinner as the largest meal of the day (β = −0.20; 95% CI = −0.36, −0.04; p = 0.01) and consuming caffeine in the evening (β = −0.16; 95% CI = −0.26, −0.05; p = 0.003) were associated with shorter sleep duration (Table 2).

Moreover, naps lasting>30 min, having dinner as the largest meal, as well as all variables related to evening screen exposure (screen time before bed, use of electronic devices to fall asleep, and sleep with screens turned on) were associated with longer latency. Evening screen exposure and napping were also associated with higher frequencies of nocturnal awakenings.

Evening alcohol consumption was associated with lower nocturnal awakenings (β = −0.15; 95% CI = −0.26, −0.05; p = 0.003), and, smoking increased in ∼12 min the sleep latency (95% CI = 5.66, 19.10; p < 0.001) (Table 2).

**Fig. 2:**
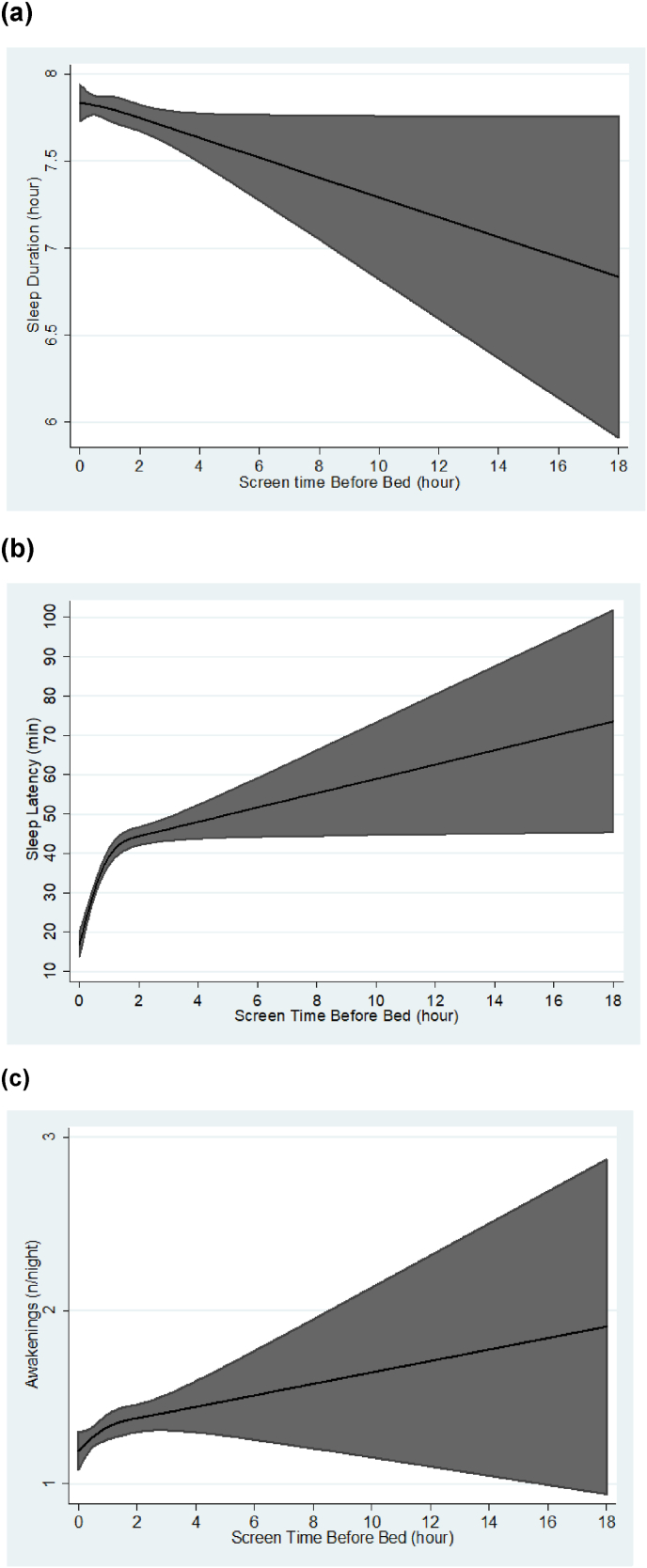
Screen time before bed related to sleep duration, latency, and nocturnal awakenings. Black lines plot the predicted sleep duration/latency/awakenings values with 95% CI (grey fill). Models are adjusted for age, sex, region, marital status, educational level, evening diet quality, and BMI. Abbreviation: CI, Confidence Interval.

Restricted cubic splines models (Fig. 2) illustrate that the negative association of screen time before bed with sleep duration (**a**) and its positive association with sleep latency (**b**) and nocturnal awakenings (**c**) were linear. The highest value of sleep duration and the lowest values of both sleep latency and awakenings were seen in the absence of the use of electronic devices with illuminated screens (time equal to zero).

## 4 Discussion

As far as we are aware, this is the first study among general Brazilian adults on the role of sleep and circadian hygiene in sleep impairments. We investigated the association of sleep-promoting and sleep-disturbing behaviors (time-of-day of physical exercise, pre-bedtime routine, naps, evening use of electronic devices with illuminated screens, reporting dinner as the largest meal of the day, evening consumption of caffeine and alcohol, and smoking) with sleep quality indicators.

Our results are partially in concordance with our hypotheses and other findings from sleep research.

### 4.1 Time-of-day of exercise

We found that morning physical exercises decreased the odds of self-reporting poor sleep quality and was associated with decreased values of sleep latency and nocturnal awakenings. However, its evening practice was not associated with sleep outcomes.

Although it is often proposed that exercising close to bedtime, especially if strenuous, may result in the release of hormones such as adrenaline, increased body temperature, and alertness, making it more difficult to relax and get restful sleep [25], the studies are inconclusive and controversial, pointing to the lack of consistent evidence to prove the positive or negative relationship between evening exercise and sleep quality [26]. Individual factors, such as exercise tolerance at different times-of-day, contribute to the divergence of results [8].

However, morning exercises, by improving the synchronization of internal biological rhythms with the natural day/night cycle, seem to benefit sleep for the majority, independent of sex, age, and chronotype [25]. Moreover, exposure to natural light during morning exercises may help to suppress melatonin, the hormone that regulates sleep and wakefulness, increasing nocturnal “sleep pressure” (homeostatic sleep drive) [25,27].

### 4.2 Pre-bedtime routine

Contrary to our hypotheses, incorporating a pre-bedtime practice (dimming the environment lights in the evening, reading books, drinking soothing tea, and incorporating integrative therapies) showed no or negative effect on sleep outcomes.

Although studies about sleep hygiene interventions to improve sleep have reported inconsistent findings [16,28], our findings must be interpreted with caution, as we do not rule out the possibility that these practices were more frequent among those with sleep problems, precisely because of the problem itself. More often than not patients, long before they seek professional help, are already aware of some popular and disseminated sleep hygiene, and so have addressed their most egregious infractions.

### 4.3 Naps

Nappers presented decreased nocturnal sleep duration and increased nocturnal awakenings and napping for more than 30 min was associated with higher odds of poor sleep quality.

According to the American Sleep Foundation's evidence-based recommendations and guidance, among adults, no napping indicates good sleep quality and, although panelists were uncertain or there was disagreement about the cut-off points for nap duration, more than 100 min does not indicate good sleep quality [24].

Also, it seems that napping longer than 30 min would have a greater impact on nocturnal sleep. This duration allows the individual to have a light sleep, without entering deeper stages of the sleep cycle (REM, Rapid Eye Movement), which would promote an increase in alertness, while longer durations would increase drowsiness and mental confusion upon waking up [29].

Also, there may be a bilateral relationship between sleeping during the day and the duration and quality of night sleep. On the one hand, short and poor sleep results in excessive daytime sleepiness, with the need for compensation throughout the day. And, in turn, napping, especially for a long time, can decrease tiredness and drowsiness at the end of the day, which would impact duration, frequency of awakenings, and sleep efficiency [30]. These behaviors must create a vicious cycle, where napping is associated with worse nocturnal sleep, and this unsuccessful sleep generates the need for daytime rest.

### 4.4 Evening diet, caffeine and alcohol consumption, and smoking

Regarding evening eating, those who reported dinner as the largest meal presented higher odds of poor sleep quality, decreased sleep duration, and increased sleep latency.

Chrononutrition-related research, which focus on the link between meal temporal patterns and metabolic disturbances [22,23] have shown that eating during the biological night and consuming a larger proportion of food/energy intake later in the day, is associated with worse sleep quality [[31], [32], [33]]. The main reported mechanism is that sleep-wake cycles and circadian rhythms are fundamentally involved in energy metabolism and related behaviors such as eating. Over the 24-h day, human physiology is organized by the central circadian clock and peripheral circadian clocks, such that wakefulness, energy intake, nutrient processing, and activity occur during the biological daytime (when levels of melatonin are low). By contrast, sleep, fasting, inactivity, and restorative processes occur during the biological night-time (when levels of melatonin are high) [34].

Among evening caffeine consumers sleep duration was shorter and sleep latency was longer and they also presented increased odds of self-reporting poor sleep quality, and smoking was associated with longer sleep latency.

It has long been widespread that the consumption of stimulant-containing foods and beverages, especially in the evening and close to bedtime [[35], [36], [37]] and smoking tobacco, known to be addictive, in addition to being a stimulant [38] affect sleep.

Caffeine is an adenosine receptor antagonist, a hormone that regulates sleep-wake cycles, and may attenuate the increase in sleep pressure during wakefulness and lead to delayed sleep initiation and more superficial sleep [[39], [40], [41]]. And nicotine stimulates the release of neurotransmitters, such as dopamine, that cause sleep disturbance (Sabanayagam et al., 2010) and, therefore, smokers may stay awake longer, have a higher frequency of nocturnal awakenings or even insomnia when compared to non-smokers [42].

Contrary to sleep hygiene recommendations, evening alcohol consumption was associated with lower frequencies of nocturnal awakenings and lower odds of poor sleep.

Despite the consensus on avoiding alcohol consumption due to its harmful effects on health and sleep, findings similar to ours have already been reported in previous studies. Also contrary to hypotheses, Freeman et al. [43] found that more hazardous drinking among women was associated with better subjective sleep quality. In another study, aimed to examine reciprocal, within-person associations between sleep and alcohol use among young adult drinkers with insomnia, participants self-reported better sleep efficiency on heavier-drinking days (driven primarily by improvements in sleep onset latency), and they reported heavier drinking following days of better sleep efficiency (driven by improvements in total sleep time), i.e. there were reciprocal associations between subjective sleep efficiency and alcohol consumption [44]. An investigation with merchant marine cadets demonstrated that alcohol consumption improved perceived sleep quality and decreased perceived latency to sleep onset while not affecting perceived sleep duration [45].

Some mechanisms have been suggested to explain these effects. For example, a study by Sharma et al. [46] demonstrates the sleep-promoting effects of acute alcohol result from an adenosine A1 receptor-mediated inhibition of orexin neurons in the hypothalamus. These neurons are known to maintain wakefulness when activated, whereas a loss of orexin neurotransmission in the hypothalamus promotes sleep. It has also been reported that acute ethanol ingestion in non-alcoholics decreases sleep latency and increases the quantity and quality of NREM (non-rapid eye movement) sleep. However, REM (rapid eye movement) sleep is suppressed during the first half of nighttime sleep and is followed by a “REM rebound” (increased REM sleep) during the second half [47,48]. Since alcohol acts as a sedative that interacts with several neurotransmitter systems important in the regulation of sleep, large amounts of alcohol before sleep lead to decreased sleep onset latency and changes in sleep architecture early in the night when blood alcohol levels are high. However, later in the night, sleep is disrupted, and the quality of sleep worsens, as the liver enzymes metabolize alcohol [49,50].

Thus, although the relationship between alcohol consumption and sleep seems to be positive, while a small amount of alcohol may shorten sleep onset, its chronic use often leads to reductions in sleep quality, increasing the risks of sleep disorders. In turn, worsening sleep quality may lead recovering alcohol-dependent individuals to relapse into seeking alcohol to help them fall asleep [47,48].

Therefore, our results should be interpreted with parsimony. It seems plausible to assume that participants who regularly consume alcohol close to bedtime and, therefore, eventually fall asleep more quickly, have the false or mistaken impression of sleeping well. Also, occasional brief awakenings or microarousals may not be noticed or remembered in the next day, precisely because of the alcohol effect.

### 4.5 Evening use of electronic devices with illuminated screens

We found a linear negative association between screen time before bed and sleep duration, and as the time use increases, sleep latency and awakenings increase as well. The longest sleep duration, the shortest sleep latency, and the lowest frequency of awakenings were observed when there was no use of screens before bedtime. Moreover, using screens for ≥2 h before bed increased the odds of poor sleep quality. Participants who reported using screens to fall asleep had higher odds of self-perceiving poor sleep quality, and they also presented increased sleep latency and nocturnal awakenings. Except for sleep duration, sleeping with screens turned on in the bedroom or sleeping environment was positively associated with all the sleep outcomes.

Such results corroborate with other studies. Rafique et al. [51] cross-sectionally investigated the effects of cell phone use on subjective sleep quality among students and concluded that using it for up to 30 min (without any type of blue light filter), after turning off the lights, resulted in poor sleep quality. Similarly, another study [52] concluded that media use close to bedtime was negatively correlated with sleep quality.

Because the circadian rhythms cycles length, or period, of the endogenous timing system, is near, but, in most organisms, not exactly 24 h, circadian rhythms must be synchronized or trained to the 24-h day on a regular basis. In most organisms, this process of entrainment occurs through regular exposure to light and darkness [14,15].

Available evidence suggests that when humans are exposed to a light stimulus in the late biological day/early biological night, that stimulus produces a phase delay shift (a shift to a later hour), and light stimuli presented in the late biological night/early biological day produce phase advance shifts (shifts to an earlier hour). So, by changing both sleep timing and the timing of hormonal secretion, sleep patterns, and quality would be strongly influenced by evening exposure to artificial light [14,15].

In addition, some studies have found that light emitted by cell phones, computers, and TV sets can increase the brain's alertness [53] and stimulate cognitive functions, which in turn would lead to delayed sleep onset and worsening quality [54], being also reported that alerting effects of light persist into sleep [55].

Another contributing factor is that social networks also encourage the rapid exchange of messages and information, encouraging users to stay awake due to the content of messages and non-stop distractions. Furthermore, the content of these media can incite reactions to stress, such as excitement, agitation, fear, and euphoria, increasing adrenaline and vigilance when the body should be preparing for rest [15,56].

Some strategies have been reported to minimize the negative impact of this type of light on circadian health, including, for example, the use of amber glasses, which reduce the incidence of blue light on the retina, digital screen filters, which block blue light and can be programmed to run from sunset and throughout the night [57]. However, although they may reduce the effects of light on melatonin secretion, they do not eliminate the aforementioned effects of the media content of these electronic devices [8].

### 4.6 Strengths and weaknesses

Our study has a few limitations, starting with the use of self-reported questionnaires which are prone to underreporting or misreporting. Also, precise questions were used to investigate sleep domains, the questionnaire specified that responses should be based on recent behaviors (last month) and, to guarantee data as close as possible to the real usual behavior, the questionnaire differentiated weekdays (work days) and weekends (free days) [58].

Finally, despite our covariate adjustment for sociodemographic, diet-related, and lifestyle traits, we recognize that a general weakness of cross-sectional studies is that the direction of the relationship, and possible pathways of causation, can only be hypothesized.

## 5 Conclusions

In summary, our results partially agreed with our hypotheses. Among sleep-promoting practices, the protective effect of morning physical exercises on sleep quality, latency, and awakenings, has been demonstrated. However, pre-bedtime practices (dimming the environment lights in the evening, reading books, drinking soothing tea, and incorporating integrative therapies) were associated with worse sleep parameters, which could reflect habits incorporated precisely because of the existence of a sleep problem, since they are socially widespread rituals.

Among sleep-disturbing practices, nappers, smokers, those who reported dinner as the largest meal, and evening caffeine consumers were more likely to have worse sleep quality parameters. The evening use of electronic devices with illuminated screens was associated with worsening sleep quality indicators.

Such findings highlight and reaffirm that proper sleep hygiene through behavior and sleep habit modification must be the best strategy for short and long-term sleep improvement. Recommendations to promote healthy sleep, and prevent, and treat sleep-related problems, with corresponding circadian health benefits, should include engaging in regular exercise, preferably, in the morning, and avoiding naps, heavy meals close to bedtime, evening caffeine consumption, smoking, and screen exposure later in the day.

The use of electronic devices close to bedtime is contrary to human biology determined by the circadian system and therefore, although it sounds challenging in modern and technological society, they should be avoided to prevent unfavorable consequences for sleep and health.

Finally, in addition to providing the first overview of Brazilian adults' sleep patterns, considering that sleep-related problems can affect all health dimensions - physical, emotional, mental, social, and spiritual - our results reinforce the importance of incorporating a careful review of the patient's sleep history as part of primary health care protocols and routine clinical evaluations, as well as including and highlighting sleep and circadian hygiene, together with other lifestyle factors, as part of public health strategies aimed at promoting health and life quality and preventing sleep-related problems. Future research might consider how interventions focused on sleep and circadian hygiene could be incorporated into primary health care and public health policies.

## CRediT author statement

Laura Castro-Santos, participated in Data curation, Formal analysis, Validation, and interpretation; drafted the manuscript, Investigation, and approved the final version. Also agreed to be accountable for all aspects of the work in ensuring that questions related to the accuracy or integrity of any part of the work are appropriately investigated and resolved, Márcia de Oliveira Lima, conceived, designed, and coordinated the research, participated in Data curation, Formal analysis, Validation, and interpretation; drafted the manuscript, and approved the final version. Investigation, Also agreed to be accountable for all aspects of the work in ensuring that questions related to the accuracy or integrity of any part of the work are appropriately investigated and resolved, Anny Kariny Pereira Pedrosa, participated in Data curation, Formal analysis, Validation, and interpretation; drafted the manuscript, and approved the final version. Investigation, Also agreed to be accountable for all aspects of the work in ensuring that questions related to the accuracy or integrity of any part of the work are appropriately investigated and resolved, Risia Cristina Egito de Menezes, designed the research, participated in Data curation, Formal analysis, Validation, Investigation, and interpretation; revised the manuscript, and approved the final version. Also agreed to be accountable for all aspects of the work in ensuring that questions related to the accuracy or integrity of any part of the work are appropriately investigated and resolved, Renan Serenini Bernardes, designed the research, participated in Data curation, Formal analysis, Validation, and interpretation; revised the manuscript, and approved the final version. Also agreed to be accountable for all aspects of the work in ensuring that questions related to the accuracy or integrity of any part of the work are appropriately investigated and resolved, Giovana Longo-Silva, conceived, designed, and coordinated the research, raised Funding acquisition; participated in Data curation, Formal analysis, Validation, and interpretation; drafted the manuscript, and approved the final version. Investigation, Also agreed to be accountable for all aspects of the work in ensuring that questions related to the accuracy or integrity of any part of the work are appropriately investigated and resolved.

## Declaration of competing interest

The authors declare no conflict of interest.
